# Genome-wide characterization of *MATE* gene family and expression profiles in response to abiotic stresses in rice (*Oryza sativa*)

**DOI:** 10.1186/s12862-021-01873-y

**Published:** 2021-07-09

**Authors:** Zhixuan Du, Qitao Su, Zheng Wu, Zhou Huang, Jianzhong Bao, Jianbin Li, Hang Tu, Chuihai Zeng, Junru Fu, Haohua He

**Affiliations:** 1 Key Laboratory of Crop Physiology, Ecology and Genetic Breeding, Research Center of Super Rice Engineering and Technology, Jiangxi Agricultural University, Nanchang, 330045 China; 2 School of Life Sciences, Jinggangshan University, Ji’an, 343009 China

**Keywords:** MATE, Expression analysis, Phylogenetic analysis, Gene function, Abiotic stress

## Abstract

Multidrug and toxic compound extrusion (MATE) proteins are involved in many physiological functions of plant growth and development. Although an increasing number of MATE proteins have been identified, the understanding of MATE proteins is still very limited in rice. In this study, 46 MATE proteins were identified from the rice (*Oryza sativa*) genome by homology searches and domain prediction. The rice MATE family was divided into four subfamilies based on the phylogenetic tree. Tandem repeats and fragment replication contribute to the expansion of the rice *MATE* gene family. Gene structure and *cis*-regulatory elements reveal the potential functions of *MATE* genes. Analysis of gene expression showed that most of *MATE* genes were constitutively expressed and the expression patterns of genes in different tissues were analyzed using RNA-seq. Furthermore, qRT-PCR-based analysis showed differential expression patterns in response to salt and drought stress. The analysis results of this study provide comprehensive information on the *MATE* gene family in rice and will aid in understanding the functional divergence of *MATE* genes.

**Supplementary Information:**

The online version contains supplementary material available at 10.1186/s12862-021-01873-y.

## Introduction

Multidrug and toxic compound extrusion (MATE) transporters constitute a class of secondary active transporters widely present in archaea, bacteria, prokaryotic and eukaryotic cells, and rely on the electrochemical potential of sodium or hydrogen ions to excrete compounds for transport activity as part of a secondary active transport mode [18]. All MATE proteins have approximately 40 % sequence similarity [13]. Currently, three-dimensional crystal structure data are available only for the bacterial NorM protein (a norfloxacin efflux protein). The NorM protein has twelve transmembrane domains, which are double-layered along the lipid plane and arranged into two bundles of six transmembrane helices (TM1-TM6 and TM7-TM12) to form a large open cavity exposed to the extracellular space [11, 19, 37].

The first MATE transporter (NorM) was identified from Vibrio paralyticus; this protein expels norfloxacin and ciprofloxacin out of cells in an energy-dependent manner [3, 29]. Mammalian MATE proteins were first identified in humans and mice (MATE1 and MATE2) [34]. Human MATE1 and MATE2 proteins are encoded by the *SLC47A1* and *SLC47A2* genes, respectively [46], which are mainly expressed in the kidney and liver [50]. Mammalian MATE protein can be used as multispecific and electron-neutral transporters of organic cations, mediating the discharge of various organic cations and cationic drugs [12, 61].

In plants, members of the MATE protein family are extremely abundant [5], and participate in regulating plant growth and development processes. These processes include transporting secondary metabolites, toxic compounds and heavy metals; regulating disease resistance; and participating in plant hormone regulation. An *Arabidopsis thaliana* seed coat color mutant (*tt12*) has been identified. This phenotype is caused by the mutation of the *TT12* gene, which is a member of the MATE family [6], and is responsible for regulating the transport and accumulation of proanthocyanidin in the seed coat cells to the vacuole [26]. The protein encoded by the barley MATE family gene *HvAAVT1* is located on the cell membrane and is mainly distributed in barley root tip epidermal cells; its substrate is citrate, which can increase resistance to aluminum [10]. The *AltSB* gene, which is involved in similar physiological mechanisms as those of aluminum tolerance, is also found in sorghum [25]. The tobacco gene *Nt-JAT1* (Nicotiana tabacum jasmonate-inducible alkaloid transporter 1) encodes a protein located on the vacuolar membrane that can regulate the synthesis and transportation of nicotine and other alkaloids, effectively avoiding the toxicity of alkaloids to tobacco [28]. Overexpression of the *ADP1-D* gene inhibited the synthesis of auxin in *Arabidopsis*, resulting in a phenotype consisting of an abnormal plant height, an increased number of lateral branches and rosette leaves and no significant apical dominance [22]. The *MATE* gene overexpression mutant *ads1-D* in *Arabidopsis* (Activated Disease Susceptibility 1) has a reduced salicylic acid content, and is a sensitive to multiple pathogens [44]. Moreover, expression of the *BIGE1* (Big Embryo 1) gene in maize accelerates leaf and root development and causes an enlarged embryo scutellum [27]. Over expression mutants of *ZmMATE884* in Zea mays resulted in lower 1000-seed weight and smaller seed length than the wild type, indicating that *ZmMATE884* gene involved in the regulation of plant seed size [56].

In rice, there are few studies on *MATE* genes, and their functions are poorly understood. The *MATE* gene *OsFRDL1* encodes a citric acid transporter that is necessary for the efficient transport of iron to the stem in the form of an iron-citric acid complex in rice [59]. Similarly, *OsFRDL4* (*Os01g0919100*) encodes an aluminum-induced citric acid transporter located in the plasma membrane of rice root cells and is one of the components of rice high aluminum tolerance [58]. Yokosho also revealed a *MATE* gene *OsFRDL2* involvement in Al-induced secretion of citrate in rice [60]. In addition, *MATE* genes also mediate the defense response of rice, and *OsMATE1* and *OsMATE2* regulate the growth and development of plants and negatively affect disease resistance [48]. *OsMATE2* also modulates arsenic accumulation in rice [32]. Transcriptomic data also show that these genes may be related to the stress response [48]. Recently, Qin et al. cloned a gene, *DG1*, that regulates rice grain filling, encoding a MATE transporter in rice. It has been illustrated that leaf-derived ABA controls rice seed development in a temperature-dependent manner and is regulated by *DG1* [36].

Members of the MATE family participate in important regulation and control of the growth and development of organisms, but related functional studies of the rice MATE family members are still lacking, and no systematic analysis has been carried out. This study used bioinformatic methods to systematically analyze the chromosome distribution, physical properties, conservation, evolution and expression patterns of rice MATE family members, providing an important theoretical basis for the functional identification of MATE family members.

## Results

### Identification of the *MATE* genes in the rice genome

Via homology searches and domain (Pfam: PF01554) prediction, 46 genes encoding specific MATE proteins were ultimately identified in the rice genome. The genes were named *OsMATE1-OsMATE46* according to their physical location on the chromosome. The length of the proteins encoded by these genes is between 370 and 598 aa, the molecular weight ranges from 39.41 to 61.65 kD, and the predicted isoelectric point ranges from 5.01 to 11.98. Most of the proteins are neutral or partially alkaline. (Table 1).

**Table 1:** Details of the 46 MATE proteins in rice

Ensemble ID	Gene name	Chromosome	Protein length (aa)	Molecular weight (Da)	Isoelectric point
*Os01t0504500*	*OsMATE1*	1	502	53543.6	5.41
*Os01t0684900*	*OsMATE2*	1	491	52984.2	7.15
*Os01t0766000*	*OsMATE3*	1	546	58346.1	9.83
*Os01t0919100*	*OsMATE4*	1	598	61652.2	9.05
*Os02t0122200*	*OsMATE5*	2	477	50490.4	8.54
*Os02t0676400*	*OsMATE6*	2	549	57831.5	7.35
*Os02t0821600*	*OsMATE7*	2	491	52508.1	5.21
*Os03t0188100*	*OsMATE8*	3	489	52964.1	7.32
*Os03t0216700*	*OsMATE9*	3	571	59963.9	8.36
*Os03t0229500*	*OsMATE10*	3	602	61512.9	7.47
*Os03t0570800*	*OsMATE11*	3	500	53838.9	7.35
*Os03t0571700*	*OsMATE12*	3	370	39408.6	5.66
*Os03t0571900*	*OsMATE13*	3	520	55795.1	5.01
*Os03t0572900*	*OsMATE14*	3	500	53622.7	6.96
*Os03t0626700*	*OsMATE15*	3	477	51833.8	8.92
*Os03t0839200*	*OsMATE16*	3	516	53507.3	7.28
*Os03t0858800*	*OsMATE17*	3	479	50620.2	10.07
*Os04t0373400*	*OsMATE18*	4	483	50960.8	7.58
*Os04t0571600*	*OsMATE19*	4	560	59186.8	6.74
*Os05t0554000*	*OsMATE20*	5	500	53486.3	7.38
*Os06t0494400*	*OsMATE21*	6	490	52046.9	6.51
*Os06t0495500*	*OsMATE22*	6	479	51117.8	7.93
*Os06t0558300*	*OsMATE23*	6	568	58865.9	7.19
*Os06t0707100*	*OsMATE24*	6	483	51956.8	6.74
*Os07t0108200*	*OsMATE25*	7	486	52845.4	6.98
*Os07t0502200*	*OsMATE26*	7	482	51372.7	8.04
*Os07t0516600*	*OsMATE27*	7	493	51871.5	8.46
*Os08t0480000*	*OsMATE28*	8	489	52549.5	6.51
*Os08t0545900*	*OsMATE29*	8	536	55666.9	8.35
*Os08t0550200*	*OsMATE30*	8	522	56172.4	7.22
*Os08t0562800*	*OsMATE31*	8	451	47495.5	5.57
*Os09t0468000*	*OsMATE32*	9	482	51985.9	8.97
*Os09t0524300*	*OsMATE33*	9	541	56434.6	7.55
*Os09t0548300*	*OsMATE34*	9	577	60065.4	9.64
*Os10t0190900*	*OsMATE35*	10	417	46361.6	11.98
*Os10t0195000*	*OsMATE36*	10	464	50215.8	8.73
*Os10t0206800*	*OsMATE37*	10	537	57722.7	6.92
*Os10t0344500*	*OsMATE38*	10	519	54761.9	8.32
*Os10t0344900*	*OsMATE39*	10	477	50734.7	8.55
*Os10t0345100*	*OsMATE40*	10	479	50790.6	8.31
*Os11t0126100*	*OsMATE41*	11	497	54675.9	6.75
*Os11t0129000*	*OsMATE42*	11	470	51570.6	8.64
*Os12t0106600*	*OsMATE43*	12	550	58374.2	5.14
*Os12t0126000*	*OsMATE44*	12	507	55443.7	6.39
*Os12t0552600*	*OsMATE45*	12	472	49684.5	8.12
*Os12t0615700*	*OsMATE46*	12	500	54101.2	6.75

### Chromosome distribution and replication pattern of the *OsMATE* genes

**Fig. 1:**
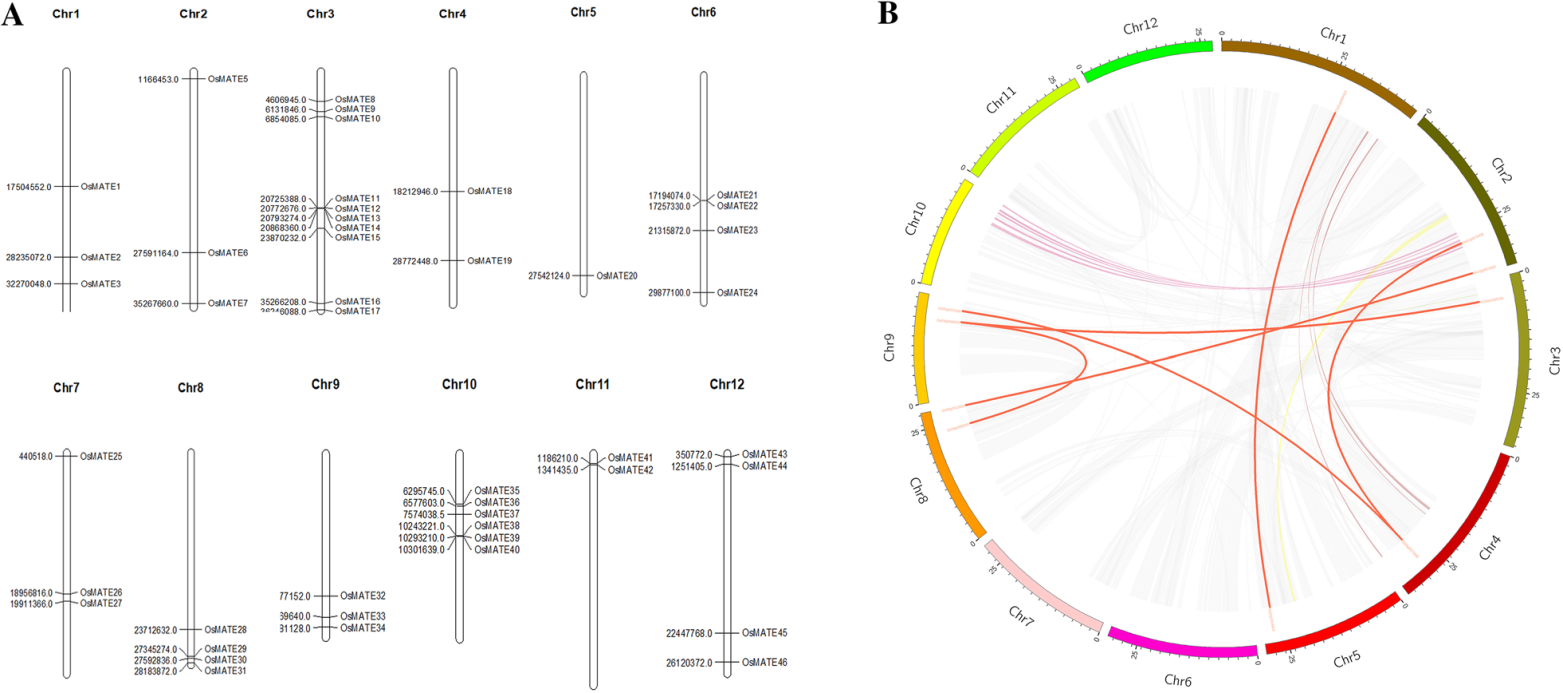
Chromosomal location and gene segmental duplication of *OsMATE.* **A** Chromosomal distribution of rice MATE genes. **B** Distribution of segmentally duplicated MATE genes in the rice genome. The segmental duplication gene pairs were linked by the lines between chromosomes

The results of the *OsMATE* gene chromosome mapping show that 46 *MATE* genes are distributed across the 12 chromosomes of rice, but the distribution is uneven. Among them, chromosome 3 contains the largest number of *MATE* genes-a total of 10, and only one of the *MATE* genes is on chromosome 5 (Fig. 1A). There are 3 pairs of tandem repeat *OsMATE* genes (*OsMATE21* and *OsMATE22*, *OsMATE39* and *OsMATE40*, and *OsMATE41* and *OsMATE42*) in rice, which are located on chromosomes 6, 10 and 11, respectively, and there is a high similarity between the protein sequences within each gene cluster. In addition, 6 pairs of fragment repeat genes were detected (Fig. 1B). Taken together, these results indicate that tandem repeats and fragment replication contribute to the expansion of the rice MATE gene family.

### Phylogenetic analysis of the MATE family

**Fig. 2:**
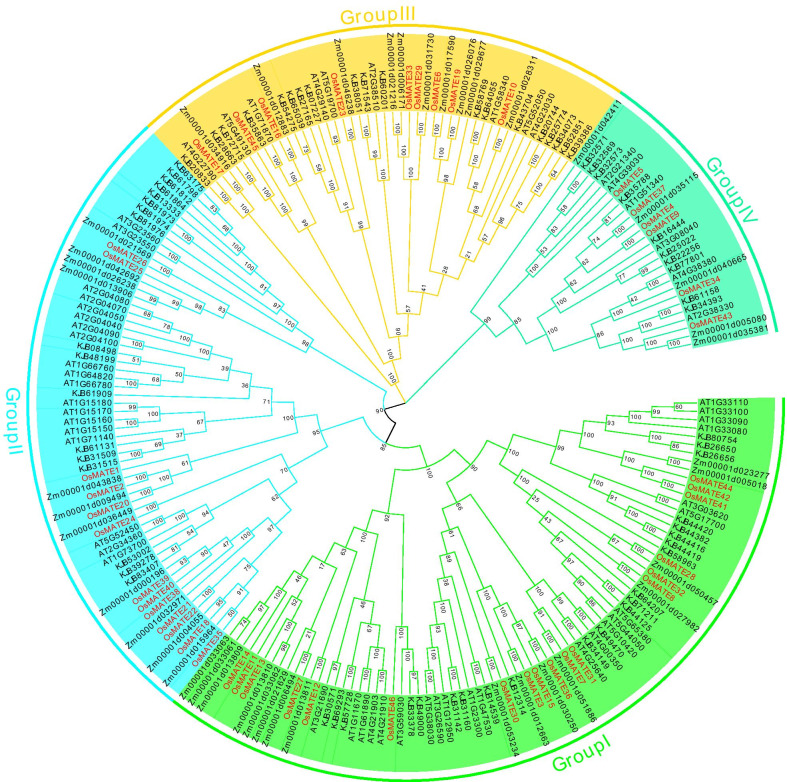
The phylogenetic tree of MATE family. The phylogenetic tree was constructed by MEGA 6.0 using the Maximum Likelihood (ML) method. Bootstrap values in percentage (1000 replicates) are indicated on the nodes. Different subfamilies are highlighted using different colors. Zm is *Zea maize*, At is *Arabidopsis*, KJB is *Gossypium *spp.

To study the phylogenetic relationship of rice MATE proteins, a phylogenetic tree was constructed using the MATE protein sequences of four different species (rice, maize, cotton and *Arabidopsis*) (Fig. 2). According to the topology of the evolutionary tree, 46 rice MATE proteins could be grouped into four groups. The first group contains the largest number of MATE proteins, a total of 18, followed by the second group, which contains 13 MATE proteins. According to the phylogenetic relationship of the protein sequences, the functions of plant MATE proteins with known functions can be used to predict the functions of rice MATE proteins.

The first group contains 18 rice MATE proteins and several known genes, including *AT3G59030* (*AtTT12*) [6], *AT4G25640* (*AtFFT*) [47] and others. The function of the known MATE transporters in this branch suggests that members of the MATE subfamily I may be involved in the transport and accumulation of plant flavonoids, anthocyanins or alkaloids. The second group contains 13 rice MATE proteins. According to genes with known functions, the members of MATE subfamily II mainly transport multiple complexes [21]. The third group includes nine MATE proteins. The members of this group of known MATE proteins have many different functions, including disease resistance, organogenesis, iron ion homeostasis regulation, and leaf senescence [4, 54]. The fourth group contains 6 MATE proteins. *OsMATE4* and *OsMATE9* have been found to participate in the secretion of citric acid in the root tips or in the transport of metal ions, indicating that these proteins are likely to participate in the physiological process of metal ion detoxification [58].

### Gene structure and conserved motifs of the *OsMATE* gene family

**Fig. 3:**
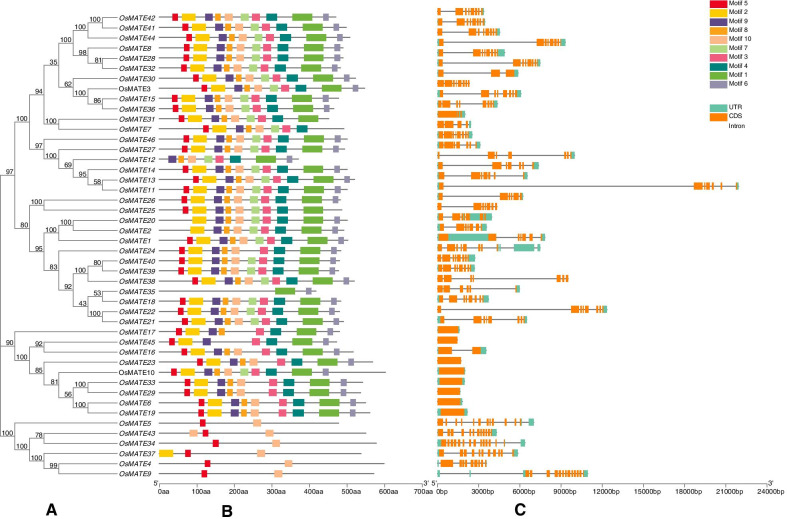
Phylogenetic relationship, gene structure and conserved motif analysis of *OsMATE* genes. **A** Phylogenetic tree of 46 *OsMATE* proteins. **B** Distributions of conserved motifs in OsMATE genes. Ten putative motifs are indicated in different colored boxes. Note: The sequence information of the ten motifs was in Table S1. **C** Exon/intron organization of OsMATE genes. Yellow boxes represent exons and black lines with same length represent introns. The upstream/downstream region of OsMATE genes are indicated in green boxes. The length of exons can be inferred by the scale at the bottom

The evolution of a family is mainly manifested as the diversity of gene structures and changes in conserved motifs. To better understand the structure of the rice *MATE* genes, the exon-intron structure of the *OsMATE* genes was analyzed using the annotation information of the rice reference genome (Fig. 3C). The *OsMATE* genes were found to contain 1 to 14 exons, which is similar to the clustering results of the evolutionary tree. Genes in the same group often have similar structures but vary in their length of introns. Most genes in group I contain 7 or 8 exons, but *OsMATE30* and *OsMATE31* contain only 3 exons; moreover, the intron length of the genes in this group varies greatly. The genes in group II have 6–8 exons, but the length of their introns is shorter than that of many genes in group I. The group III genes have the fewest number of exons, with only 1 or 2, and the length of the exons is longer than that of the members in the other three subgroups. The group IV genes have the largest number of exons (7–13). Similar results were also found in other studies [17, 42]. It has also been found that exon-intron patterns within the same phylogenetic classification group showed great similarity. This may be the result of replication of these sequences, which may also prove that the classification results are reliable.

The MEME online prediction tool was used to identify the conserved motifs in the rice MATE proteins (Fig. 3B). A total of 10 conserved sequences (motifs 1–10) were identified. The results showed that all the rice MATE proteins contained at least 2 conserved motifs, and most MATE proteins (54 %) contained all the conserved motifs. Most proteins in subfamilies I, II, and III contain similar types and numbers of conserved motifs, but there are significant differences from the proteins in the fourth group. The MATE proteins in the fourth group contained only 2 to 3 conserved motifs, and the number of motifs was significantly lower than the number of proteins in the first three groups (Fig. 3B). These findings are similar to the prediction results of the conserved motifs of the MATE proteins in soybean [23], which may indicate that the function of the protein in the fourth group is more differentiated than that of the other three groups of members.

### Characterization of putative *cis*-regulatory elements in the promoter regions of *OsMATE* genes

**Fig. 4:**
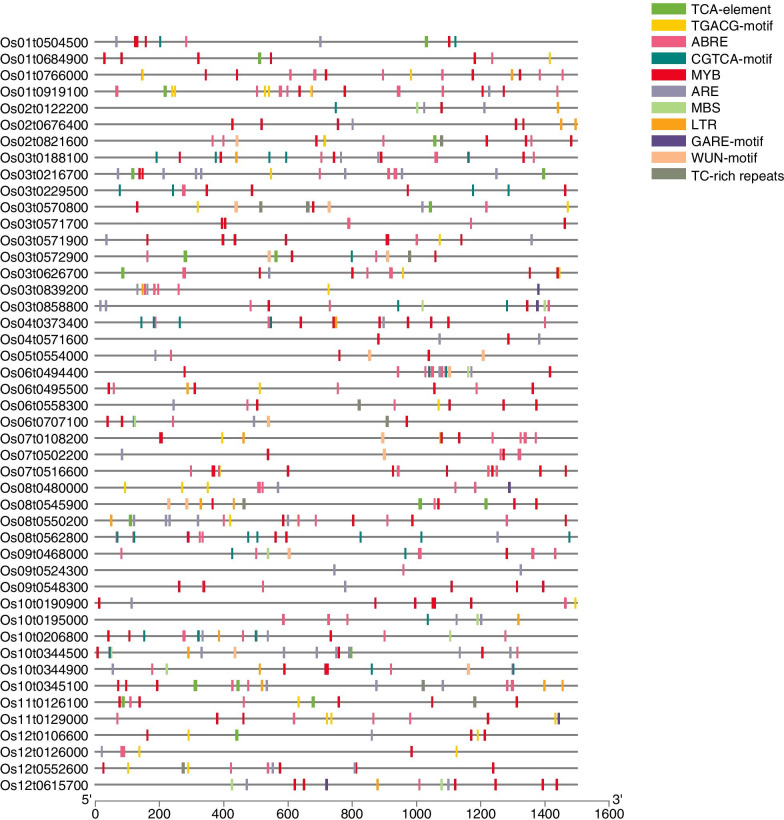
Predicted *cis*-regulatory element in *OsMATE* promoters

*Cis*-regulatory elements in the promoter regions play important roles in the plant response to stress. Using the PlantCARE database, we identified 11 putative stress-responsive *cis*-regulatory elements 1500 bp upstream of these *OsMATE* genes, including ABREs (ABA-responsive elements), TGACG motifs, CGTCA motifs (which are involved in the MeJA response), LTRs (low-temperature-responsive elements), MYBs, MBSs (MYB-binding sites), TCA elements (which are involved in salicylic acid responsiveness), TC-rich repeats (defense- and stress-responsive elements), WUN motifs (wound-responsive elements), GARE motifs (gibberellin-responsive elements) and AREs (anaerobic-responsive elements). The elements associated with the highest number of stress response elements within the *OsMATE* gene family are abscisic acid stress-related regulatory elements (ABREs) and drought-related elements (MYBs, MBSs) (Fig. 4). ABA is synthesized mainly in response to drought and high salinity stress. Among the elements, the number of defense- and stress-related response elements (TC-rich repeats) is the smallest. In addition, there are regulatory elements related to anaerobic stress (AREs), low-temperature response elements (LTRs), and hormone response elements (TGACG motifs, CGTCA motifs, GARE motifs and TCA elements, etc.). Most of the proteins in subfamily I, II and III contain similar types and numbers of conserved motifs, but are significantly different from the fourth group of proteins. The 6 MATE proteins in the fourth group only contain 2 to 4 conserved motifs, the number of motifs is significantly less than the proteins in the first three groups, and they only contain 3 motif types. It may reflect that the function of the protein in the fourth group is more differentiated than the other three groups. These results showed that the rice MATE genes and stress-related response elements are relatively complete, but the type and number of stress-related elements contained in each MATE gene promoter differ, indicating that members of the rice MATE gene family can respond differently to different stresses.

### Expression patterns of *OsMATE* genes in different tissues

**Fig. 5:**
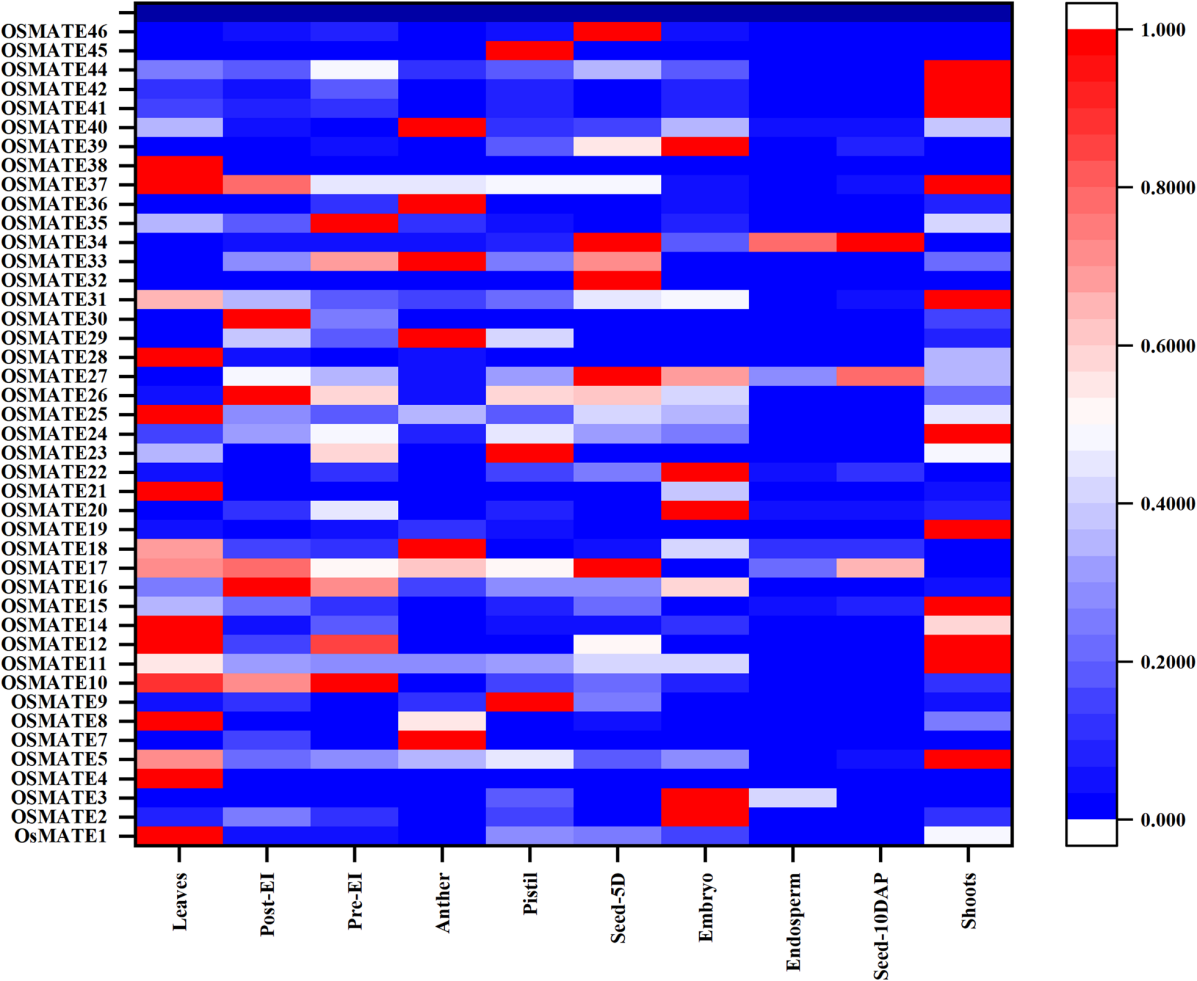
Expression pattern of rice *MATE* genes. Expression level is expressed by color and intensity: dark red indicates highest expression level, dark blue indicates lowest expression level. Other colors represent intermediate levels of expression

Via RNA-seq data, heat maps of 43 *MATE* genes represented by FPKM values in different tissues and organs were constructed. A heatmap of gene expression was generated from a representative sample of 10 different organs (Fig. 5). All *OsMATE* genes were expressed, while a few (*MATE4*, *MATE32*, and *MATE38*) were expressed only in one tissue or organ. The number of gene families with members expressed in the leaves is the largest. Eleven genes, *MATE1*, *MATE5*, *MATE14*, *MATE20*, *MATE24*, *MATE25*, *MATE26*, *MATE31*, *MATE34*, *MATE37* and *MATE40*, were expressed in all of the tissues and showed constitutive expression. Some *OsMATE* genes showed similar expression patterns in various tissues. *MATE41*, *MATE42* and *MATE44*, which were placed in the first group in the phylogenetic analysis, showed relatively high expression levels in the shoots. *MATE2* and *MATE39* of the second gene family in the phylogenetic analysis were highly expressed in embryos, and *MATE19* and *MATE45* exhibited high expression levels in the pistil. The expression in different plants parts is closely related to the functions of genes.

### Expression analysis of the *OsMATE* genes in response to abiotic stress

**Fig. 6:**
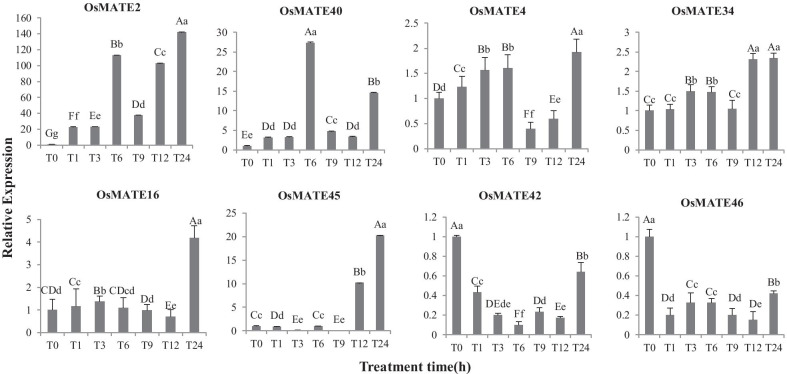
Expression pattern of genes after salt stress. Eight *OsMATE* genes representing four subfamilies were randomly selected and their relative expression in different periods was verified by qRT-PCR. The gene before treatment was used as an internal control, and its relative expression was one. All data were normalized. T0 represents before stress treatment. T1, T3, T6, T9, T12 and T24 represent 1, 3, 6, 9, 12 and 24 h after stress treatment, respectively. T The expression levels were represented by mean ± SE. Error bars were obtained from three measurements. Small letters and capital letters above the bars indicated significant differences (p < 0.05, Duncan) and (p < 0.01, Duncan) respectively between different time stages

**Fig. 7:**
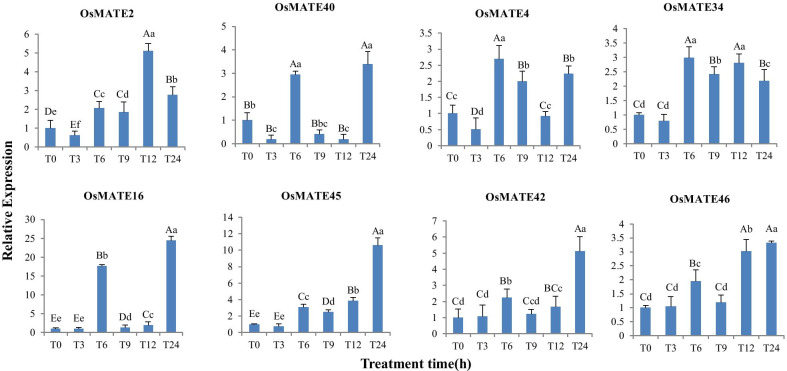
Expression pattern of genes after drought stress. Eight *OsMATE* genes representing four subfamilies were randomly selected and their relative expression in different periods was verified by qRT-PCR. The gene before treatment was used as an internal control, and its relative expression was one. All data were normalized. T0 represents before stress treatment. T3, T6, T9, T12 and T24 represent 3, 6, 9, 12 and 24 h after stress treatment, respectively. The expression levels were represented by mean ± SE. Error bars were obtained from three measurements. Small letters and capital letters above the bars indicated significant differences (p < 0.05, Duncan) and (p < 0.01, Duncan) respectively between different time stages

Crop production and yield quality in most farmlands are severely affected by salt and drought stresses. To further explore the expression changes in *MATE* genes in response to various abiotic stresses, including salt and drought, we randomly used eight *OsMATE* genes from the four phylogenetic groups. qRT-PCR was used to measure the transcript levels of the *OsMATE* genes. The expression levels of the MATE genes under salt and drought stresses varied among the eight members (Figs. 6 and 7). *MATE42* and *MATE46* were downregulated after treatment. The remaining *OsMATE* genes were upregulated under salt stress, but the changes were not as extreme as those under drought stress. The expression of four genes (*MATE2*, *4*, *16* and *45*) reached the highest level for 24 h after salt stress, but the expression of two genes increased sharply for 3–6 h after salt stress. The *OsMATE* genes were sensitive to drought stress, with none being downregulated. Notably, the expression level of all genes increased significantly for six hours after drought treatment. There were different responses and regulatory mechanisms of the MATE family members under various abiotic stress conditions.

*MATE2* and *MATE4* belong to the first group, and their expressions increased sharply 6 h after salt stress. The expressions of *MATE4* and *MATE34* in the second group did not change significantly after different treatment times. The expression levels of *MATE16* and *MATE45* were lower in the early stage of salt treatment and drought treatment, and reached the peak 24 h after treatment. The expression of two genes in the fourth group, *MATE42* and *MATE46*, decreased after salt stress.

## Discussion

The *MATE* gene family comprises one of the largest family of genes that encode transporters in plants, and the members are involved in many physiological processes during plant growth and development. At present, the structure and function of the members of the MATE transporter family have been analyzed in many plant species, such as *Arabidopsis* [6, 21], soybean [23], tomato [42], and sesame [17]; however, this information has been less reported in rice [15].

The MATE family of genes has a large number of stress response elements, including elements that respond to low temperature, drought [31], mechanical damage and hormones, and it has been shown that stress-related elements in the promoter regions of plants under adverse conditions can improve gene transcription and enhance the resistance of plants to adverse conditions [30, 35, 57].

Many functional gene families evolve and expand during gene replication. The MATE gene family has expanded mainly through tandem and segmental duplication events [45, 54]. In contrast to the amplification pattern of the *MATE* gene family in tomato, tandem replication in the *OsMATE* gene family is less abundant. In rice, there was more fragment replication of the *MATE* genes, similar events occurred in the diploid cotton and soybean. Fragment replication was the main driving force for the amplification of the *MATE* gene family, which may be related to the multiple genome-wide replication events experienced by the genome during the evolutionary process.

Phylogenetic analysis is one of the most commonly used methods to predict gene function, which can provide a relatively reliable reference for further functional verification. In this study, by clustering with known plant MATE proteins, rice MATE proteins were divided into four groups, and most of the MATE proteins with different functions were grouped in different groups. These results are similar to the *MATE* gene classification of other species [23]. Some of the genes in the first group were highly expressed in the roots. The proteins in group I are mostly involved in the transport and accumulation of various plant secondary metabolites. For example, *AtTT12* (*At3g59030*) encodes a protein that is localized on the vacuolar membrane and is involved in the transport of proanthocyanidin precursors into the vacuole, and the seed coat color of the mutant is lighter than that of the wild type [6]. The AtFFT protein is a flavonoid transporter that affects the level of flavonoids in *Arabidopsis* [47]. In other species, MATEs have similar functions. *MtMATE1* is the key gene whose encoded protein is involved in transmembrane transport of original anthocyanins [62], and the protein encoded by *MtMATE2* mediates vacuolar sequestration of flavonoid glycosides and glycoside malonates in *Medicago truncatula* [63]. *Nt-mate1* and *NT-MATe2* in tobacco mediate the transport and isolation of nicotine in the vacuole [49], and *MdMATE1* and *MdMATE2* of apple were grouped with *AtTT12* in the phylogenetic tree. Both genes were transferred into *tt12* mutants of *Arabidopsis* to rescue the mutant phenotype to that of the wild type, indicating that *MdMATE1* and *MdMATE2* are the key genes involved in proanthocyanidin transport in apple [9]. Similarly, in grape, *VvMATE1* and *VvMATE2* regulate the transport of procyanidins during berry development [38].

Proteins in the second group mediate the transport and efflux of multiple complexes and toxins. For example, *AtALF5* (*At3g23560*) is associated with plant resistance to toxins in *Arabidopsis*. *Alf5* mutants are unable to form lateral roots, and their roots are more sensitive to multiple complexes than are wild-type roots [7]. *AtDTX1*(*At2g04040*), the first cloned plant *MATE* gene, has been identified as encoding a detoxifying efflux carrier for plant-derived antibiotics and other toxic compounds, including Cd^2+^ [21]. *OsMATE2* in rice is upregulated in response to arsenic stress and predominately in developing seeds, regulating the transport and accumulation of arsenic in grains [32]. Tobacco *NT-JAT1* has been shown to encode a nicotine transporter that is involved in the deposition of alkaloids in the vacuole [28]. Some genes in the second group are highly expressed in embryos and may be involved in the regulation of seed development.

The third group contained many known genes involved in a variety of physiological processes, including disease resistance, organogenesis, ion transport and leaf senescence. For example, *AT4G29140* encodes activated disease susceptibility 1 (*ADS1*), a putative MATE transport protein that negatively regulates plant disease resistance and the elongation of hypocotyl cells [41]. *ZF14* encodes a plant MATE transporter that is localized to the Golgi complex and other small organelles and is involved in determining the rate of organ initiation; this gene is also involved in iron homeostasis when plants are under osmotic stress [4]. *ELS1* is related to the senescence of *Arabidopsis* leaves [54]. *Bige1*(*Zm00001d012883*) encodes a trans-Golgi apparatus-related transporter that is involved in regulating organ structure and size. Loss of Bige1 function leads to increased embryo size and accelerated production of lateral organs, including both leaves and roots, as well as early flowering [27]. RNA sequencing also showed that some of the genes in the third group were highly expressed in the pistil and are involved in Fe nutrition during organ initiation and development.

In the fourth group, the MATE proteins are known to be involved in metal ion transport. *Arabidopsis* EDS5(enhanced disease susceptibility 5), which is a chloroplast- localized salicylic acid transporter, is an essential component of salicylic acid-dependent signaling needed for disease resistance and expression induced by salicylic acid [33, 43]. MATE transporters transport iron by transporting citric acid and protocatechuic acid, which can chelate Fe ions and increase their solubility. OsFRDL1 (Os03g0216700) is a citric acid transporter localized in pericycle cells and is necessary for the effective transport of iron to the stem in the form of iron-citric acid complexes [59]. FRD3 is likely to function in root xylem loading of iron chelators or other factors necessary for efficient iron uptake from the xylem or apoplastic space and into leaf cells [39, 40]. OsFRDL4 is an aluminum-induced citric acid transporter localized in the plasma membrane of rice root cells and is one of the components involved in the high aluminum tolerance of rice [58]. Two MATE proteins, OsPEZ1 and OsPEZ2, were identified in rice as being essential to achieve Fe ion exudation by transporting phenolic compounds and protocatechuic acid [2, 16].

The gene structure analysis showed that the members of each group had similar exon-intron structures, but the gene structure of the members in the different groups were quite different, indicating that the members of the rice *MATE* gene family had differentiated into groups with different functions during the evolutionary process. The conserved base sequence prediction results also showed that rice MATE proteins in the different groups have different types and numbers of conserved motifs. In the first, second and fourth groups, MATE proteins contain a relative abundance of conserved motifs, which explains why the role of these transporter variations is relatively large. Substrate proteins may exert a variety of different functions and were present in the three groups of known plants; these proteins include MATE proteins involved in secondary metabolite transport and accumulation of a variety of external complexes, ion transport and organs, and a variety of physiological functions. The third group had relatively few exons. In some studies, to respond to stress in a timely manner, genes must be activated quickly, aided by a compact structure with relatively few introns [24, 64]. These properties were the same as those of the *MATE* genes in both cotton and maize[24, 64]. QRT-PCR clearly indicated that *OsMATE* genes play a significant role in tolerating the effects of salt and drought stress in rice. The response pattern is different, which shows that MATE genes have different regulatory pathways in response to abiotic stress.

## Conclusions

Overall, in the present study, genome-wide analysis of 46 *MATE* genes identified in the rice genome and the comparison with homologous genes of other species revealed the potential function of these genes in transport. Given their important role in plant physiology, MATE transporters may be ideal targets for breeding programs to improve agricultural-related traits such as aluminum tolerance, iron nutrition and accumulation of secondary metabolites (such as increasing anthocyanin contents or eliminating toxic alkaloids).

## Materials and methods

### Identification of MATE transporters in the rice genome

Rice genomic data were downloaded from the Ensemble database (http://asia.ensembl.org/index.html). Using the sequence of the fifty-six *Arabidopsis* MATE protein sequences as the query sequence, BlastP was used to search the MATE family genes from the whole genome database of rice. The sequence of the conserved domain of the MATE proteins was determined via a hidden Markov model (HMM) (PF01554). We downloaded the HMM profile of MATEs from the Pfam protein family database [8], and used it as the query (P < 0.001) to search the rice protein sequence data. After removing the redundant sequences, a total of 46 MATE family genes were identified. The NCBI CDD (https://www.ncbi.nlm.nih.gov/Structure/bwrpsb/bwrpsb.cgi), Pfam database and SMART database [20] (http://smart.embl-heidelberg.de/) were used to confirm the conserved domain. The theoretical isoelectric point (pI), the number of amino acids and the molecular weight (MW) of the MATE proteins were computed by ExPASy (http://web.expasy.org/protparam/)  [55].

### Chromosomal locations and gene duplication analysis

The chromosomal locations of the *OsMATE* genes were illustrated by MapChart software 2.2 [51]. Segmental and tandem duplication events of the MATE family were identified using the Multiple Collinearity Scan tool kit (MCScan) [53] from the Plant Genome Duplication Database. Homologous genes (E < 1e-40, similarity > 50 %) within 3 gene loci were considered tandem replication genes, and TBtools software was used to draw a schematic diagram of the positional relationship of the collinear genes.

### Phylogenetic analysis of MATE proteins

Phylogenetic analysis was performed using the full-length sequence of MATE amino acids from *Arabidopsis*, maize and cotton combined with the sequence of the newly identified OsMATE proteins. Multiple sequences were aligned by ClustalX software, with the default parameters. MEGA 6 software with 1000 bootstrap tests was used to construct an unrooted neighbor-joining phylogenetic tree.

### Gene structure and motif analyses

Gene structure analysis was performed using the Gene Structure Display Server [14] (GSDS, http://gsds.cbi.-pku.edu.cn/) program, with the default settings. Motifs within the MATE proteins were identified using MEME [1] (http://meme-suite.org/), with the default settings (motif width: between 6 and 50 (inclusive)). The maximum number of motifs was 10.

### Analysis of *cis*-regulatory elements in the promoter regions of *OsMATE* genes

The 1.5 kb upstream sequence of the *MATE* gene translation initiation codon was downloaded from the Phytozome database. Using the PlantCare database (http://bioinforma-tics.psb.ugent.be/webtools/plantcare/html/), *cis*-regulatory elements in the 1500 bp upstream region were subsequently predicted.

### Analysis of RNA sequencing (RNA-seq) data from the MATE family

RNA-seq data were downloaded from the Rice eFP Browser (http://www.bar.utoronto.ca/efprice/cgi-bin/efpWeb.cgi) to study the expression in various tissues and at various stages of reproductive development of rice. To render the data suitable for cluster display, the absolute FPKM value was divided by the average of all the values and then log2 transformed. Excel 2010 was used to perform data filtering and analysis. Origin software was used to generate a heatmap.

### Plant materials and abiotic stress treatments

Changhui 121, a cultivated rice variety from Jiangxi province and bred by Jiangxi Agricultural University, was selected in the experiment. The mature seeds were placed in the petri dish, sterilized by 2 %NaCLO, soaked at 30 ℃ for 48 h, and then placed in a perforated PCR plate. A PCR plate was separated by placing 24 seeds as biological repeats. The treatment and control groups were repeated with two plates. All seedlings were placed in the growth chamber with 14-h day/10-h night photoperiod and a 26/24 °C (day/night) temperature cycle. Rice seeds were cultured in sterile water in first 3 days and then in Yoshida nutrient solution.

For salt stress, rice seedlings were cultivated in an artificial growth chamber to the three-leaf stage. Yoshida nutrient solution with a final NaCl concentration of 120 mM was used for stress treatment. For drought stress, the plants were treated with 15 % PEG-6000 at 29 days after rice germination. Under these different stress conditions, the aboveground parts of whole plants were collected at 0, 3, 6, 9, 12 and 24 h after treatment and immediately frozen in liquid nitrogen and stored at − 80 °C.

### RNA extraction and expression analyses of *MATE* genes

In different stress treatments, three plant leaves with the same growth potential were taken for RNA extraction. Total RNA was extracted using an RNA Simple Total RNA Kit (Takara, Japan). First-strand cDNAs were synthesized using a PrimeScript First Strand cDNA Synthesis Kit (Takara) in a total of 20 µl reaction volume consisting of 1 µg of total RNA, 4 µl of 5X Prime Script RT Master Mix, and RNAase-free ddH2O. The PCR program was as follows: 95 °C for 2 min followed by 40 cycles of 95 °C for 5 s and 60 °C for 30 s.

Quantitative real-time PCR was performed on an ABI 7500 quantitative real-time PCR system following the manufacturer’s instructions. Primer Premier 5 was used to design primers specific to the *MATE* genes (Additional file 1: Table S2). The *Actin* was used as a reference gene. QRT-PCR was performed in a final volume of 20 µl, consisting of 2 µl of cDNA, 10 µl of 2X SYBR Green Master Mix (Takara), and 1 µl of forward and reverse primers. The amplification program was as follows: initial denaturation at 95 °C for 5 min; 40 cycles of denaturation at 95 °C for 10 s and annealing at 60 °C for 20 s; and a final extension at 72 °C for 20 s. Three biological replicates were performed, and three technical replicates were performed per cDNA sample. The relative expression values were calculated by 2 ^−ΔΔCT^ method.

## Supplementary Information

**Additional file 1.** The supplementary information of genome-wide characterization of* MATE* gene family and expression profiles in response to abiotic stresses in rice.

## Data Availability

The datasets generated and/or analysed during the current study are available in the fishare repository, [https://figshare.com/articles/online_resource/the_date_of_MATE_family_rar/14616717].
