# Supplementary material for: Genome-wide characterization of MATE gene family and expression profiles in response to abiotic stresses in rice (Oryza sativa)
Source: BMC Ecol Evol. 2021 Jul 9;21:141. doi: 10.1186/s12862-021-01873-y (PMC8268253; doi:10.1186/s12862-021-01873-y)
Supplement: Supplementary file 1 — Additional file 1. The supplementary information of genome-wide characterization of MATE gene family and expression profiles in response to abiotic stresses in rice. [file 12862_2021_1873_MOESM1_ESM.docx]

**Additional file**

**Table S1** **The sequence information of the ten motifs in Figure 3C**

| **name** | **the sequence information of motifs** |
| --- | --- |
| Motif 1 | SLLPJLAVSILLBGIQPVLSGVARGCGWQKLVAYVNLGAYYLVGIPVAVLLAF |
| Motif 2 | VTGYSLLLGMASALETLCGQAYGAKQYHMLGVYLQRS |
| Motif 3 | CLEWWYYEILVLLAGLLPBPE |
| Motif 4 | MIPVGLGAAISVRVSNELGAGRPRAARFA |
| Motif 5 | VSQAFVGHLGELELA |
| Motif 6 | RTBWEKEAEKAKERV |
| Motif 7 | CPETWTGFSMEAFRDLGAFLK |
| Motif 8 | FPLQKFLQAQSIVLP |
| Motif 9 | GQDPEIAAAAGAYARWLIPQL |
| Motif 10 | HVPLSWLLVYKLGLGLAGAAL |

**Table S2 Primers for qRT-PCR in this study**

| **Gene name** | **forward primer(5’→3’)** | **Reverse primer(5’→3’)** |
| --- | --- | --- |
| Actin | GATGACCCAGATCATGTTTG | GGGCGATGTAGGAAAGC |
| MATE2 | AGCCTCCTCTTGGAATGG | GAAAACCCATAGAAGTGATAGA |
| MATE4 | AAATTAATCCCTGTCGACGAG | GCCACCATCTGTTTCTGCT |
| MATE16 | TCCTCCTCGCCTACATCC | GTGACCACCTCGTACCACC |
| MATE34 | TGACGCGAACGATTCTACC | CTACAGGACCTAAACGACCAA |
| MATE40 | GCTACAGCCTCCTCACGGG | AGCGACGGGAGCATCCAC |
| MATE42 | CGTCACCGTCATCAGCCA | CAAGCAGGATGCCATTGC |
| MATE45 | CTCACCCATTGAACATCTC | TTGTTTGTACCTCGGACA |
| MATE46 | GGCTCATGGTCTGGACTC | CCAACGATCACTCACGCT |
